# An Essay on Delirium Tremens

**Published:** 1846-06

**Authors:** Edwin Ramsey Long

**Affiliations:** U. S. Army


### ILLINOIS AND INDIANA

### MEDICAL AND SURGICAL JOURNAL.

Vol. I. NEW SERIES.—JUNE, 1846. No. 2.

### PART I.—ORIGINAL COMMUNICATIONS.

### ARTICLE I.

*An Essay on Delirium Tremens.* By Lieut. EDWIN RAMSEY LONG, M. D., U. S. Army.

[The following Essay, left in manuscript by our lamented friend, whose obituary appeared in our last No., though unpretending in its character, is intrinsically worthy of an attentive perusal by all, and will be especially acceptable to his numerous friends, who may wish to possess a memorial of one, estimable both as a companion, and an enthusiastic votary of science.

In it are expressed his views upon the nature and treatment of Delirium Tremens—the results of numerous observations made upon this disease, during his long and honorable official connection with the army of the United States, and, though made previous to his becoming a member of the medical profession, the conclusions, as will be seen, harmonize fully with views entertained by many of our most experienced and scientific men. W. B. H.]

*Delirium Tremens.*—As there has existed, and does still exist, a great diversity of opinion in the Medical Profession, as to the pathology and treatment of this disease, I deem it an appropriate subject for some speculations of my own. Although I have not the presumption to believe that I can set at rest the various mooted questions involved, yet, perhaps, I may at least, by a careful examination of the subject, be able to expose some unfounded theories which have obtained some repute, and which, unfortunately, have in some instances given rise to very injudicious treatment. In order to do this I shall make a sketch of the various phenomena exhibited in the course of the disease; of the several modes of treatment that have been most in vogue; and of the facts brought to light by autopsical researches.

In considering the several modes of treatment, in connection with its true pathology, we will be forcibly struck with the wide difference of opinion, entertained by men of undoubted ability, of great scientific acquirements, and long experience in the treatment of this malady. And it is not asserting too much, to say that there are few maladies which exhibit in their history and treatment, so much empiricism and professional dogmatism; or which so forcibly exemplifies the folly of "prescribing for names instead of diseases." While one asserts that it is a disease of simple debility, requiring no other treatment than simple stimulants, another affirms—not so, but we must view it as a case of nervous irritability, calling for the specific use of opiates. Another declares that it is nothing more nor less than an acute inflammation, and should be treated accordingly; and a fourth gravely tells us that it requires no treatment at all, but if let alone will heal itself. How are these conflicting opinions to be reconciled? Are they the opinions of men whose standing in society entitles them to respect? To satisfy ourselves on this point, we have only to refer to the standard text books of Medical Schools. But why so great a diversity of opinion? Is the disease of such a mysterious character as to elude the scrutiny of the laborious investigations that have from time to time been bestowed upon it? Have the theorists based their speculations upon facts discovered by faithful post-mortem examinations? I am far from believing that all of these were empirical in their practice, although it cannot be denied, that among a vast number of the profession, opium has been as much regarded as a specific for delirium tremens, as quinia for febrile intermittents. But I think the great error has been in individuals basing their theory and practice solely upon their own observation and experience, instead of availing themselves of the more extended observations of the profession at large. For instance, we hear of one that has made many post-mortem examinations of the victims of this disease, who has invariably found unequivocal evidence of acute gastritis,—he concludes that this is the true character of the disease, and in future directs his treatment to this peculiar lesion of the stomach. Another has made as many examinations and finds the same conclusive evidence of cerebritis or meningitis, and of course recommends another treatment. A third has seen many cases, terminating fatally, which did not exhibit a single vestige of organic lesion. Notwithstanding these opposing views, I am of the opinion, that upon taking a more general view of all the facts developed by modern pathology, that we can in some measure reconcile them to each other, notwithstanding the conflicting and opposing deductions that have been drawn from them. And if we are not so fortunate as to determine with certainty the true pathology of the disease, upon which to base a rational course of treatment, we, at least, may be able to say what it *is not*, if we cannot precisely say what *it is;* and to establish some landmarks, which, if they serve not to point out with exactness the true channel to the adventurer, may admonish him of the localities of certain quicksands that might endanger his craft, or prejudice the interest of his proprietors. And it may not be amiss here to observe, that notwithstanding the inestimable advantages derived from the labors of late pathologists by the enlightened practitioner, and while we would recommend to every one, to regard this branch of his profession as the most faithful pilot to direct him through the labyrinths of theories and speculations, through which he must inevitably pass in his pursuit of truth, yet it would be hazardous for any one to assert whether the treatment of delirium has been benefitted or not, by the disclosures of morbid anatomy, from the fact before referred to, that many have predicated theories upon their own observations, instead of those made in common by the profession. But let us proceed to our investigation. "Delirium tremens (says Eberle) is a variety of mental disease characterized by inquietude, tremors, continued watchfulness, cool skin, perspiration, delirious loquacity, and sensorial illusions." Again he says: "The disease commences with lassitude, general indisposition, a feeling of *distress in the epigastrium*, nausea and vomiting, a sense of confusion in the head, a want of sleep, and anxious expression of countenance."

"So long as the customary quantity of stimulus is taken, it seldom or never supervenes, but if from sickness, or temporary disgust, the ordinary potations are left off, the activity of the brain becomes morbidly increased, and mental disorders, in many instances arise." "It is important to bear in mind, (says Dr. Coates,) that the disease is the result, not of the application, but of the sudden intermission of these articles. At a certain stage of the disease the patient begins to manifest mental disorders, become loquacious, says he feels well, and is tormented with a continued succession of various alarming, disgusting, and ludicrous apparitions.

"When the disease supervenes upon a pleurisy, or other inflammatory affection accompanied with pain, the principle disease seems to disappear, even to the eye of the experienced practitioner, to be reproduced in a later period, when the brain and nerves regain their ordinary tranquility. The pulse in this disease, varies considerably in different cases. In some instances it is *hard*, *full*, and *frequent;* but much more commonly, soft, full, and quick, without strength or tension. The tongue is humid, and covered with a white fur. The *bowels* are *torpid*, and there is usually a *loathing of food* through the whole of the disease, but the *thirst for cold drinks* is *almost always* considerable. It appears to be generally conceded that the disease has its primary and essential location in the sensorium commune, and is wholly independent of inflammation of this organ. It would seem to consist of a purely dynamic disorder—a morbid activity of the brain, from the sudden abstraction of ordinary stimulus, by which its excitability had been long depressed or blunted." Dr. Coates considers it as consisting of a heightened activity of the sensorium, from a generation of an inordinate activity of the brain.

Dr. Klapp has published a number of cases with observations, going to show that the proximate cause or seat of the disease is *in the stomach.* It is asserted that dissections show that traces of previous inflammation existed in the stomach, and nearly in all instances, nausea, vomiting, and foul tongue occur, and that the operation of an emetic brings off a viscid, light brown fluid, of the *consistence* of *tar*, from the stomach, and that finally, this disease yields more easily and frequently under this treatment than any other. "With regard to the foul tongue and other evidences of gastrites, Dr. Coates found them generally absent." "Dr. Sutton mentions them only as symptoms accompanying the disease, when it occurred in connection with other diseases." Dr. Stokes says, "you have all seen cases of delirium tremens, but perhaps are not aware that it may arise under two opposite classes of causes. In some cases a patient who is in the habit of taking wine or spirituous liquors every day in considerable quantities, meets with an accident, or gets an attack of fever. He is confined to his bed, put on an antiphlogistic treatment, his liquor stopped, and an attack of delirium tremens comes on, and symptoms of high cerebral excitement appear. Another person not in the habit of frequent intoxication, takes to a fit of drinking, and is attacked with delirium tremens. In the first case, the delirium arises from the want of the accustomed stimulus —in the second the disease is different, and consequently, in this view, it would be a manifest departure from sound practice, to treat both cases alike. In the first variety, when the disease arises from the want of the accustomed stimulus, there is no doubt that patients have been cured by the administration of the usual stimulus. Indeed, this seems the best mode of treating this form of disease. But is it proper or admissible in the second variety, when the disease is caused by an occasional excess in the use of ardent spirits, yet in many cases, a man who has been attacked by delirium tremens after a violent debauch, is ordered a quantity of porter, wine, brandy, or opium, and the worse he gets the more is the quantity of stimulants increased. Let us consider what the state of the case is. A large quantity of stimulant liquors have been taken into the stomach, the mucous surface of that organ is now in a state of intense irritation, the brain and nervous system in a highly excited condition. Are we to continue this stimulation? What would be the obvious result? Increased gastritis, encephalitis, or menengitis. This supervention of an inflammatory condition of the brain is not understood by many physicians. They go on administering stimulant after stimulant, totally unconscious that they are bringing on decided cerebral inflammation. I have witnessed the dissection of a great many persons who died of delirium tremens, and one of the most common results of the dissection was unequivocal marks of inflammation, both in the *stomach* and *brain.* But there have been cases in which no distinct mark of gastric inflammation could be discovered. In all cases, however, when the delirium supervenes on an excessive debauch, there is more or less of gastritis, and though a patient may occasionally recover under such circumstances, under the stimulant treatment, yet I am convinced the physician will frequently do harm by adopting it. This complication of gastritis, is exceedingly curious in another point of view, as it illustrates how completely the local symptoms are placed in abeyance, and as it were, lost during the prevalence of the strong sympathetic irritation. The patients abdomen may not be tender, the tongue may not be red, the symptoms present may be indicative of cerebral affection, and yet a gastric inflammation be going on all the time, and all the appearance of cerebral disease be removed by treatment calculated to subdue an acute gastrites. Is all this theory? No. For we have practised on this principle of treatment with extraordinary success in the Meath Hospital; we have seen violent outrageous cases of delirium subside by the application of a few leeches and internal use of iced water, without a single drop of laudanum. On the other hand, when a stimulating plan of treatment was employed, and the patient died, we have most commonly found inflammation in two places—in the stomach, and in the brain or its membranes. The rule, then, is, in a case of delirium from the want of the customary stimulus, use the stimulant and opiate treatment; but when it comes on after an occasional violent debauch, such remedies must be extremely improper. Adopt here everything calculated to remove violent gastric irritation."

With regard to blood-letting, an eminent author informs us that when it has been principally relied on, he has observed a fatal termination of the disease in almost every case. Dr. Armstrong has known, in cases where the constitution was not shattered by repeated intoxication, early and moderate blood-letting of much use. Another says emetics deserve more attention as curative means in delirium tremens, than other remedies that have been employed, with the exception of opium. "Cupping about the head, may, under some circumstances prove useful." "In an instance, I, (Dr. Eberlie) attended about six months since, when there was turgescena of the head, and a state of delirium approaching raving phrenitis, immediate and decided benefit was derived from cupping."

"Blisters will also do good when applied to the legs, in cases attended with violent cerebral excitement."

From the foregoing extracts which set forth the views of some of the most eminent authors, relative to the pathology and treatment of delirium tremens, I make the following deductions, viz: 1st. In *every* case there is found a peculiar morbid state of the sensorium commune, either dynamic or adynamic, manifested by delirium, tremors, sleeplessness and mental illusions. 2d. In a vast proportion of the cases, decided evidences of gastrites, or other more serious lesions of the stomach, such as ulceration, scirrhus, &c., are found.

3d. That in other cases, where there is no marks of inflammation or ulceration of the stomach, there is noted a marked derangement of the digestive apparatus, such as foul tongue, nausea, &c.

4th. Not unfrequently traces of active inflammation of the brain, or its meninges, have been developed by autopsical researches.

5th. Torpor and impacted state of the bowels are not unusual, they being filled with feculent matter, and irritating secretions.

Such, then, being the prominent features of the disease, what is its pathology or essential nature? To determine this we must proceed on the principle of exclusion. It is, very obviously, an organic lesion of the stomach, or other part of the alimentary canal,—of the brain, or a peculiar morbid excitement of the nervous system, independent of inflammation in any tissues of the body.

Commencing with the first of them, we have it well authenticated, that cases of this disease which have terminated fatally, have been observed where no evidences of inflammation of the stomach was to be found, the same may be said of the intestines, and the brain, with its meninges; hence we conclude that the disease does not necessarily involve a lesion of either of these organs. But is there a case on record, in which the peculiar morbid excitement of the nervous system, as manifested by signs just enumerated, was not prominently and *invariably* presented? If not, are we not warranted in the conclusion, that this is the pathognomonic trait of the disease, without which it has no existence. All the other most prominent and common associations, may be wanting, and still these phenomena are found, the former are not therefore essential parts of the disease, but may, and frequently do accompany it. For, although it may be said that a case of delirium tremens, in which they are not found is extremely rare, yet *one* well established case, is of itself sufficient to demonstrate that these lesions are not essential to the disease; we therefore assume that the malady is essentially one of the nervous system.

We are now ready to consider the various methods of treatment that have been most commended. In view of the facts that we have just exhibited, we have a disease of the nervous system, but almost invariably complicated with inflammation of the brain or stomach, with torpidity and loaded condition of the intestinal tube, for which, some have recommended the exclusive use of stimulants, others, opiates, others again, emetics, and a fourth class, equally strenuously advocating the antiphlogistic plan, not applying one or the other according to the varied symptoms of the case, but *one* and alone, single handed, decrying all others as useless or decidedly pernicious. Such practice is too absurd to need comment. If instead of pursuing this course, we adopt the more rational one of discarding all empiricism, and selecting remedies according to the varied features of the case, we will find that it is no difficult matter to determine upon a plan of treatment that will not disappoint our expectations in the result. In the first place, if we meet with a simple uncomplicated case of delirium tremens, as the disease is located in the nervous system, we have only to resort to such remedies as experience has found to be most effectual in controlling nervous symptoms, such as opiates, camphor, and the like. But as we seldom meet with such cases, but find them most usually connected with organic lesions of other tissues, we must primarily direct our attention to the investigation of these. By refering to the foregoing extracts we see cerebritis, gastritis, and impacted bowels are the most frequent morbid associations, and consequently our first object is the removal of them, using for this purpose those remedial agents that are best suited to accomplish these ends.

As gastritis is the *most* common complication, with a full abdomen, we should make it a primary consideration to relieve the latter without aggravating the former. For this reason we should not use cathartics, but give the preference to enemata, inasmuch as cathartics will inevitably increase the irritability of the stomach, and if inflamed, may occasion an incurable lesion of that organ. But in the event of there being a full, hard, tense pulse, bleeding should never be omitted, and if, superadded to this, there are marks of a strong determination of blood to the head, cups, or leeches will be advisable. Venesection, if practised, should precede other remedies. Should, however, a foul tongue, nausea, and other symptoms manifest a general derangement of the digestive organs, and no indication of gastritis be found, an emetic will do essential service. Having, then, premised venesection and emesis, when warranted by the symptoms just given, we will find no remedy so effectual as a moderately stimulating injunction, by bringing away the vitiated matters lodged in the intestinal tube, which, perhaps, more than any other contingent disorder, tends to keep up the nervous irritability and mental hallucinations. Having cleared the bowels, we should next address the appropriate remedies to the stomach, to subdue any inflammation that may be indicated, such as iced water and leeches, bearing in mind, however, that we use not this, nor any other remedy without a special indication by present symptoms, unless, perhaps, we have reason to suspect the existence of such disease that is masked by the predominant power of cerebral inflammation, and in this malady, as gastritis, is not unfrequently covered by inflammation of the brain, it may not be amiss to use some counter irritants or revulsions to the stomach, as a precautionary remedy.

After removing by suitable remedies, all contingent or accidental disorders that we may find connected with the primary malady, which, as we have before stated, seems to be undoubtedly located in the nerves, we may now venture to administer such remedial agents as we have found to be most effectual in controlling morbid derangements of these tissues, such as opium, camphor, asafœtida, &c., and if we have been successful in the removal of what may be termed the collateral or contingent disorders, we will find that the primary or nervous malady will readily yield to this treatment, and our patient will soon fall into a refreshing sleep, that may be regarded as a favorable crisis of the delirium.

A moment's consideration of the several modes of treatment that have found most favor with practitioners, will show us at once why they have sometimes been successful, and at others totally inefficient or decidedly prejudicial. The truth is, that each of them is good in its place, and no one of them always applicable. It would be just as absurd to use opiates or stimulants invariably in all cases of this disease, as it would be to apply the like remedies to all cases of remittent fever, and precisely for the same reasons. We must discard all notions of empiricism, and be governed by established maxims of therapeutics, as much in this as in other maladies.

We are surprised to see that even Dr. Stokes seems to think that there are only two remedies necessary in this malady, one to remove gastritis, the other, the class of stimulants applicable to an opposite condition of the system. Now I contend, that there are cases where it would not be proper to use either of them; for instance, when the bowels are torpid and full of vitiated accumulations, what effect would iced water and leeches, to the stomach, on the one hand, or enormous doses of opium, on the other, have in subduing the nervous excitability? This is a case calling for enema. Hence we see the propriety of selecting, in every case, such remedies as are indicated by present symptoms.

From the foregoing we conclude, that not the stimulating—the bleeding—the purging—or the antiphlogistic treatment is to be recommended *singly* or exclusively, but *each* or *all* of them as circumstances may render necessary and proper.

